# Defoaming and Toughening Effects of Highly Dispersed Graphene Oxide Modified by Amphoteric Polycarboxylate Superplasticizer on Oil Well Cement

**DOI:** 10.3390/ma17112523

**Published:** 2024-05-23

**Authors:** Min Zeng, Yubing Xing, Yongxu Xie, Dawei Xu, Xia Miao, Jintang Guo

**Affiliations:** 1 Sinopec Research Institute of Petroleum Engineering Co., Ltd., Beijing 102206, China; zengmin.sripe@sinopec.com (M.Z.); xudw.sripe@sinopec.com (D.X.); 2 School of Chemical Engineering and Technology, Tianjin University, Tianjin 300350, China; xyb_9@tju.edu.cn (Y.X.); yongxuxiexyx@163.com (Y.X.); 3 Zhejiang Institute of Tianjin University (Shaoxing), Shaoxing 312300, China

**Keywords:** cement toughness, oil well cement, modified nanomaterials, electrostatic self-assembly, defoamer, graphene oxide

## Abstract

The aggregation of graphene oxide (GO) during the hydration process limits its wide application. Polymer superplasticizers have been used to improve the dispersion state of GO due to their adsorption and site-blocking effects, though the formation of a large amount of foam during the mixing process weakens the mechanical properties of cement. A highly dispersed amphoteric polycarboxylate superplasticizer-stabilized graphene oxide (APC/GO) toughening agent was prepared by electrostatic self-assembly. Results demonstrate that the APC/GO composite dispersed well in a cement pore solution due to the steric effect offered by the APC. Additionally, the well-dispersed GO acted as an antifoaming agent in the cement since GO nanosheets can be absorbed at the air–liquid interface of APC foam via electrostatic interactions and eliminate the air-entraining effect. The well-dispersed APC/GO sheets promoted cement hydration and further refined its pore structure owing to the nucleation effect. The flexural and compressive strength of the cement containing the APC/GO composite were enhanced by 21.51% and 18.58%, respectively, after a 7-day hydration process compared with a blank sample. The improved hydration degree, highly polymerized C-S-H gel, and refined pore structure provided enhanced mechanical properties.

## 1. Introduction

In recent decades, the toughening of cement-based composites has always been on the cutting edge of research and a key target in their engineering application due to the serious problem of brittle cracking. The characteristic properties of cement lead to a weak flexural strength, poor durability, and high susceptibility to cracking, which significantly shorten the service life of cement. A basic strategy to enhance crack resistance requires modifications at the nanoscale since most cement damage can be attributed to its chemical and mechanical structural flaws [1]. Nanomaterials prevent and/or delay cement cracking by filling the region between calcium silicate hydrate (C-S-H) gel layers and refining micro-/nanoscale defects. Reinforcing mechanisms include crack bridging/deflection, CNT pull-out, and filling and nucleation effects [2]. Graphene oxide (GO) is a graphene derivative composed of several layers of folded two-dimensional carbon sheets with various oxygen-containing functional groups on the surface or between the sheets [3]. As a nano-reinforcing material used in various engineering matrixes, the large specific surface area, high modulus, and reactivity of GO make it a promising material for enhancing the toughness of cement [4,5] and concrete composites [6]. More importantly, GO can disperse in water due to its plentiful oxygenated hydrophilic functional groups, thereby accelerating the hydration process of cement and forming effective interfacial bonds between hydration products [7]. During the hydration process, GO sheets can act as active sites to accelerate the deposition and growth of hydrated crystals, thus providing GO-reinforced cements with good mechanical properties [8,9]. The carboxyl groups of GO sheets are prone to cross-linking with Ca^2+^ in alkaline cement paste [10,11], which leads to the immediate agglomeration of GO in cement and weakens its dispersion effects, resulting in a decrease in its reinforcing effects on cement. 

To improve the working efficiency of GO via better dispersion, ultrasonic techniques [12,13] were applied. However, once an ultrasonic treatment is stopped, nanomaterials often reunite [14]. Also, serious agglomerations of GO still occur in cement pore solutions due to the complexation of Ca^2+^ [12]. To inhibit the cross-linking of GO and Ca^2+^ in pore solutions, the chemical modification of GO nanosheets has aroused significant interest among researchers [15,16]. Zhang [17] grafted GO to a polymer via RAFT polymerization to improve the dispersion state of GO sheets in cement. Some researchers have stated that fly ash or SiO_2_ [18,19] could be used as a template to aid in GO dispersion through the chemical coating method, but the laborious, low-yield, and time-consuming chemical modification process makes it difficult to conduct large-scale industrial-grade applications of this process in practical projects. 

Recently, large amounts of surfactants, such as sodium dodecyl sulfate [20], ethylene-vinyl acetate [21], or polycarboxylate superplasticizer [22], have been used to disperse GO. Surfactants can be employed to increase the workability of cement slurry and disperse nanomaterials and cement grains [23,24]. Zhao [25] used polycarboxylate superplasticizer (PC) to modify GO to improve its dispersibility and the mechanical properties of cement composites. However, the utilization of surfactants as superplasticizers or dispersants in cement slurry has been limited as some surfactants can cause foaming and the harmful pores formed in the cement stone would reduce the compressive strength of the cement [26]. The addition of a defoamer is usually desired to reduce and hinder foam production [27]; however, the defoamer can further complicate the cement system [28]. We urgently need to improve the dispersion of nanomaterials and overcome the air entrapment problem caused by surfactants.

Herein, an amphoteric polycarboxylate superplasticizer (APC) was synthesized to disperse GO during the cement hydration process. The prepared APC was anchored to the surface of GO, mainly by electrostatic self-assembly, and the steric hindrance of the APC’s side chains helped the GO disperse well. The well-dispersed GO can also be used as a defoamer to eliminate the harmful macropores formed due to the air entrapment effects of the APC and further improve the mechanical strength of the cement. The dispersion state of the APC/GO composite in a pore solution was characterized by microscopy techniques. The defoaming effects of GO on the APC samples were mainly investigated using a pore structure analysis. The hydration process of the APC/GO-modified cement was explored using an isothermal calorimeter. The mechanical properties of the APC/GO-modified cement and their working mechanisms were investigated by thermal gravimetry (TG) and ^29^Si nuclear magnetic resonance (^29^Si NMR). This study confirmed the defoaming properties of GO using experiments, laying an experimental foundation for further explorations of the defoaming mechanisms of nanomaterials. The incorporation of the APC/GO composite significantly improved the mechanical properties of oil well cement, offering a suitable approach for the mechanical improvement of GO-modified cement composites with potential for widespread application in construction cement and concrete.

## 2. Experiment

### 2.1. Materials

GO was provided by Suzhou Tanfeng Graphene Technology Co., Ltd. (Suzhou, China). The APC polymer was fabricated by radical polymerization using ω-hydroxy polyethylene glycol ether (HPEG, Mw = 2400 g/mol, Deruike Chemical Co., Ltd., Tianjin, China), dimethyldiallyl ammonium chloride (DMDAAC, Luyue Chemical Co., Ltd., Taian, China), and acrylic acid (AA, Jiangtian Chemical Co., Ltd., Tianjin, China). The molar ratio of HPEG: DMDAAC: AA monomers was 1:3:3, and the initiator was ammonium persulfate. Class G oil well cement from Jiahua Special Cement Co., Ltd. (Leshan, China) was used in this research. The chemical composition of the cement was measured using X-ray fluorescence (XRF), the results of which are displayed in Table 1.

### 2.2. Modification of GO with APC

GO was subjected to ultrasonic vibration to achieve randomly dispersed flakes. The APC was then incorporated into the dispersion phase of the GO via continuous stirring for 10 min to obtain an APC/GO composite. Subsequently, the APC/GO composite was examined using Raman, X-ray diffractometer (XRD), and zeta potential measurements. A pore solution was fabricated [29] to represent the chemical environments of the cementing components during the early cement hydration process which were used to evaluate the dispersion performance of the GO-NS and GO/NS composites in cement slurry. A 3D digital microscope (VHX-2000, KEYENCE, Osaka, Japan) was utilized to observe the agglomeration at a high multiplication. The hydrodynamic size distribution of APC/GO in pore solution was tested via dynamic laser scattering (DLS) technique and GO agglomerates using laser diffraction (LD) technique.

### 2.3. Preparation of Cement Specimens

According to API Recommended Practice 10B-2, pure cement and cement pastes containing GO, APC, the APC/GO composite, and an APC/D composite, respectively, were prepared. The mixing designs for the pastes are exhibited in Table 2, and the water-to-cement ratio was 0.44:1. The cement pastes were cured at 60 °C for 1 day, 3 days, and 7 days to test their compressive and flexural strengths.

### 2.4. Characterization

The cement hydration process was measured by isothermal calorimetry, using a cement hydration heat tester (HD-SHR-08Z, Tianjin Huida, Tianjin, China). Moreover, samples for hydrated product analyses (Brunauer-Emmett-Teller (BET), Mercury Intrusion Porosimeter (MIP), TG and ^29^Si MAS NMR) were derived by milling a cement stone and sieving it using a 200-mesh sieve. BET and MIP tests were carried out using an automatic specific surface area and porosity analyzer (TriStar 3000, Micrometrics, Livermore, CA, USA) and a mercury porosimeter (AutoPore V 9600 Micrometrics, Livermore, CA, USA). In addition, the size distribution of the pore structures in the cement specimens was examined by optical image analysis. ImageJ was used to measure the size of every pore in the cement specimens. The hydrated crystals were analyzed using a TG test (TG 209F3, Netzsch, Selb, Germany, heated from 35 °C to 800 °C at 10 °C/min under N_2_) and a ^29^Si MAS NMR spectrometer (JNM ECZ600R, JEOL, Akishima, Japan, using an 8 mm HXMAS Probe, νR = 6 kHz, a relaxation delay of 8 s, and 600 scans).

## 3. Results and Discussion

### 3.1. Characterization of APC Dispersant

**Figure 1:**
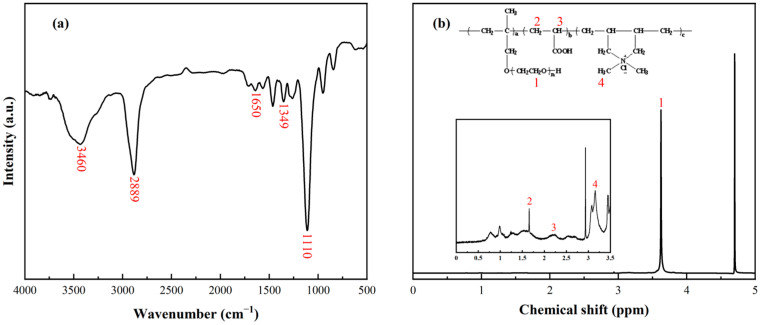
(**a**) FTIR spectrum of APC dispersant. (**b**) ^1^H-NMR spectrum of APC dispersant.

The chemical structure of the APC dispersant was investigated using FTIR and ^1^H-NMR spectra. In Figure 1a, the broad band at 3460 cm^−1^ and the sharp peak at 2889 cm^−1^ originate from the stretching vibration bands of the hydroxyl (-OH) and methyl group (-CH_3_), respectively. The sharp peak presented at 1110 cm^−1^ represents the asymmetric stretching vibration of ether groups (-C-O-C) [30]. The peak at 1349 cm^−1^ corresponds to the -CN stretching vibration of quaternary ammonium groups (–NR_3_^+^), and the adsorption band around 1650 cm^−1^ results from a carbonyl group (-C=O), indicating the presence of both cationic and anionic groups in the APC.

As shown in Figure 1b, the ^1^H-NMR spectrum was utilized to analyze the APC. The prominent peak at 4.70 ppm results from the deuterium in the solvent D_2_O, and the sharp peak located at 3.5–3.8 ppm corresponds to the chemical shift of protons on the ether bond (-CH_2_-CH_2_-O-) in the side chain of the APC dispersant. In the inset in Figure 1b, two small peaks located around 1.60 and 2.27 ppm prove the existence of methylene (-CH_2_-) and methylene (-CH-) on the polymer backbone chain. The peak near 3.27 ppm is ascribed to -NCH_3_, further confirming the composition of the APC.

### 3.2. Structural Characterization of APC/GO Composite

**Figure 2:**
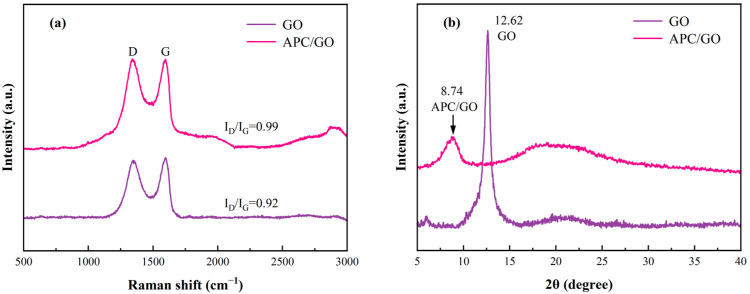
(**a**) Raman spectra of GO and APC/GO composite. (**b**) XRD spectra of GO and APC/GO composite.

Raman spectra is one of the most effective tools for characterizing carbon-based materials as it is highly sensitive to electronic structures. In Figure 2a, GO presented typical bands at 1344 and 1593 cm^−1^ which correspond to the D and G bands and are assigned to crystal defects introduced by oxygen functional groups and the in-plane vibration of sp^2^ carbon atoms, respectively [31]. Generally, the intensity ratio of the D and G bands (I_D_/I_G_) expresses the defect density in graphene [32]. After the introduction of the APC, the I_D_/I_G_ exhibited an increase from 0.92 (GO) to 0.99 (APC/GO), suggesting the presence of amorphous polymers on the GO surface and the successful modification of the APC.

The variation in the XRD curves of the GO and the APC/GO composite was also checked, as illustrated in Figure 2b. The GO exhibited a characteristic sharp peak around 2θ = 12.62°which was attributed to the (001) plane, and its interlayer spacing was 0.70 nm owing to the intercalation of oxygen functional groups in the graphite sheets [33]. Compared with the GO, the APC/GO composite had an obvious left shift and a reduction in the intensity of its diffraction peaks. Meanwhile, the interlayer spacing of the APC/GO composite was correspondingly further increased to 1.01 nm because the adsorption of the APC introduced long side chains to the surface of the GO, resulting in stronger steric hindrance between neighboring GO layers.

The surface charges of the APC/GO composite and GO were measured in deionized water using the zeta potential to verify their electrostatic interactions. The zeta potentials of the aqueous-phase GO and APC/GO sample were −44.5 ± 1.3 mV and −19.7 ± 0.55 mV, respectively. The hydrolysis of numerous -COOH, -OH, and other oxygenated groups on GO sheets causes the GO to have a negative charge in the aqueous phase [34]; hence the absolute zeta potential of the aqueous-phase GO phase was much higher, while the positive charge of the -NR_3_^+^ in APC neutralized the negative charge of the GO layers, leading to a reduced absolute zeta potential. This experiment revealed efficient electrostatic interactions between the APC and GO, suggesting that the GO was functionalized by the APC through electrostatic self-assembly.

### 3.3. Dispersion Behaviors of GO and APC/GO Composite

**Figure 3:**
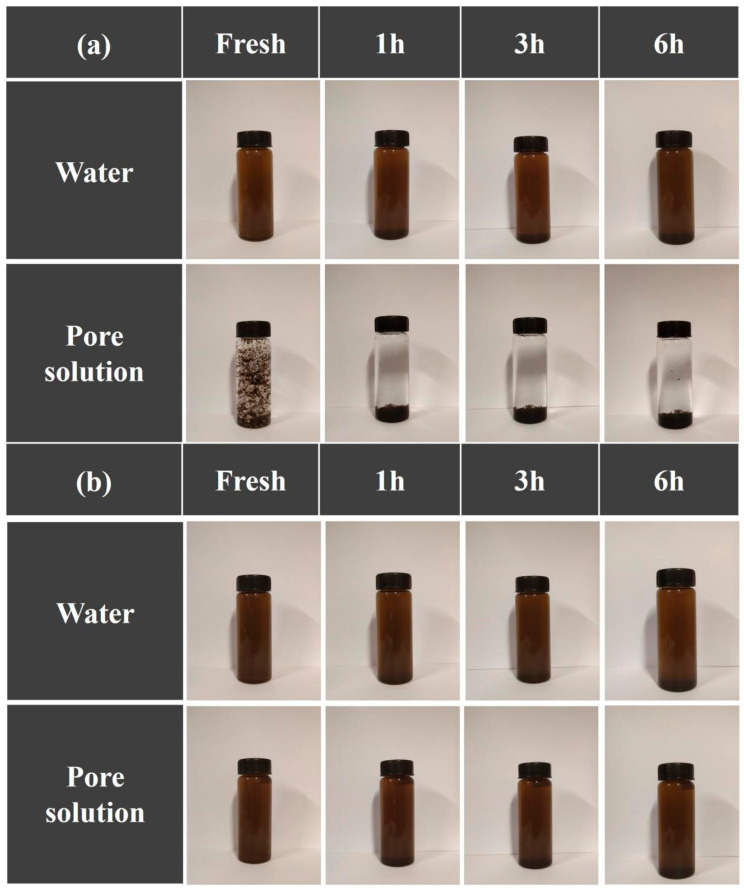
Dispersion states of (**a**) GO and (**b**) APC/GO in various environments.

Direct visual observations of the GO’s dispersion state in various solutions are presented in Figure 3. As shown in Figure 3a, the high content of hydrophilic oxygen groups allowed the GO to disperse stably in water, while its aggregation and deposition can be observed in the pore solution. Abundant Ca^2+^ in the pore solution interacted with the oxygen functional groups on the GO sheets, bridging the edges of the GO sheets, intercalated among the carbon base surface, and formed hydrogen bonds between the oxygen-containing groups of the GO and the interlayered water molecules [35]. Except for the cross-linking of Ca^2+^, GO can react with OH^−^ from highly alkaline slurry, and the oxygen-containing groups will be reduced [36]. The electrostatic repulsion originating from the negatively charged oxygenated groups is lost, and the GO sheets are prone to aggregating.

In Figure 3b, no discernible agglomeration was observed at the macroscopic level for a few hours, indicating that the APC maintained a stable dispersion of GO in both water and the pore solution. The -NR_3_^+^ in the APC backbone chain would allow for efficient electrostatic attraction with the oxygen-bearing groups on the GO and hydrogen bonding by -COOH in the APC [30], both of which ensured the assembly of the APC on the GO’s surfaces; thus, the long-sided chains of the APC provided sufficient steric hindrance between the GO sheets.

**Figure 4:**
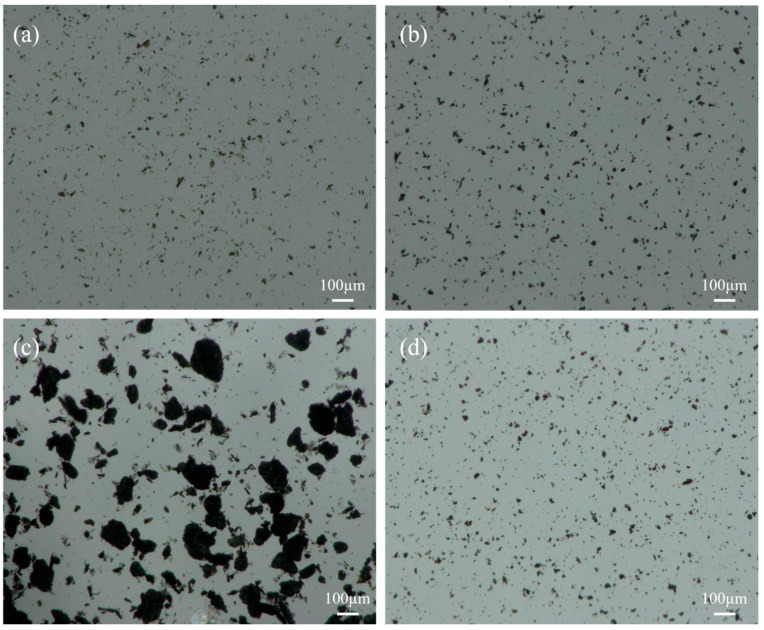
Microscopic dispersion states of (**a**) GO in water, (**b**) APC/GO composite in water, (**c**) GO in pore solution, and (**d**) APC/GO composite in pore solution.

The microscopic dispersion states of the GO and APC/GO composite in different environments were further investigated using a 3D digital microscope. The GO and APC/GO compound both presented a homogeneous and steady dispersion in water, which indicates that the great dispersion of GO in water was not impaired by introducing the APC. Corresponding to the discernible sediment in Figure 4a, the GO showed poor dispersion and many GO aggregates, ranging from a few microns to 100 microns, congregated in the pore solution. By comparison, the APC/GO composite maintained a relatively high level of dispersion in the pore solution.

**Figure 5:**
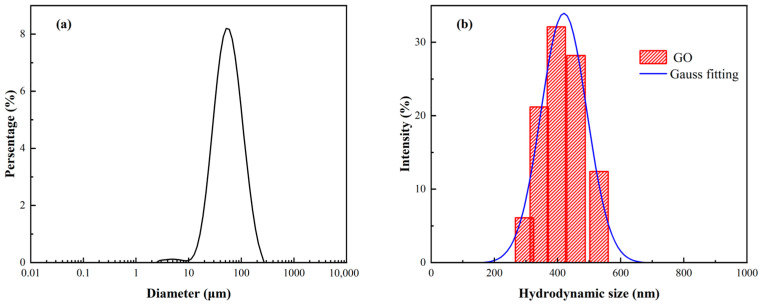
Size distribution of (**a**) GO aggregates and (**b**) APC/GO nanosheets in pore solution.

The size distributions of the GO and APC/GO composite in the pore solution were further determined using the laser diffraction method and dynamic laser scattering (DLS) method [37], respectively. As shown in Figure 5a, the median diameter of the GO aggregates in the pore solution was 58.7 µm. To obtain the size distribution of the GO and APC/GO composite, the DLS approach, which has been widely applied to determining the hydrodynamic size of 2D materials, was selected [38,39]. As demonstrated in Figure 5b, the APC/GO aggregates in the pore solution were substantially smaller than the GO aggregates. The average diameter of the APC/GO nanosheets in the pore solution was mainly distributed about 420.3 nm, indicating that the GO can maintain good dispersion in the pore solution after its modification with the APC.

Based on the observed results, the surface-modifying effects of the APC on GO can improve the dispersion state of GO during the hydration process. The physical adsorption of the APC/GO composite occupied the -COO^−^ groups of the GO and inhibited its cross-linking with Ca^2+^. The APC assembled on the GO nanosheets through the electrostatic attraction between its cationic group -NR_3_^+^ and the -COO^−^ on the GO surface, and the space steric hindrance from a side chain with ether groups (-CH_2_CH_2_O) on the APC further ensured the dispersion stability.

### 3.4. Dispersion State of APC/GO in Cement

**Figure 6:**
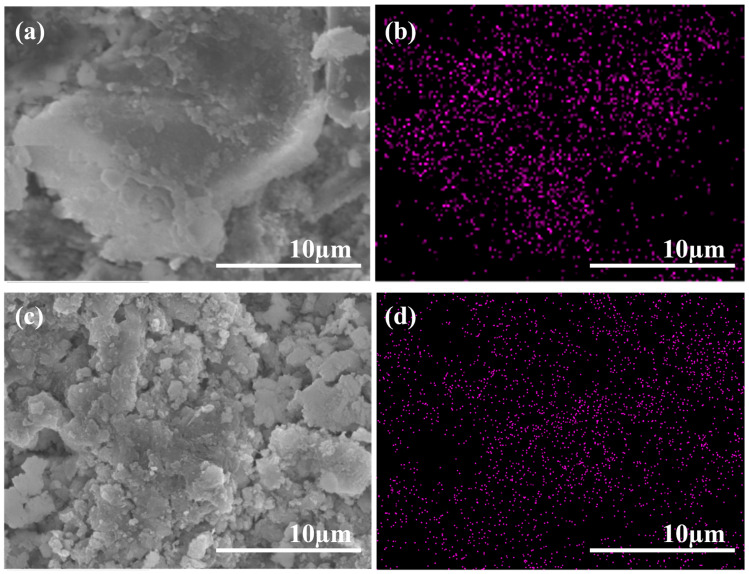
SEM images of GO (**a**) and APC/GO composite (**c**) modified cement; corresponding C elemental maps of GO-modified (**b**) and APC/GO-modified (**d**) cement.

Figure 6 shows the microstructures and SEM-EDS mapping of the cement samples modified by GO and APC/GO. Figure 6a reveals a folded and wrinkled GO structure with poor dispersion. To further verify the dispersion of GO in cement, an EDS analysis for the C element was conducted on the SEM images. Figure 6b demonstrates that the C element distribution in the GO sample is uneven and significantly enriched. The poor dispersion of GO in cement without the APC aligns with the adverse agglomeration findings in the pore solution. On the contrary, in the samples containing the APC/GO composite, C elements were found to be uniformly distributed (Figure 6d), suggesting that GO did not agglomerate in the cement. Hence, the APC/GO sheets were well dispersed in the cement, Figure 6c, and could act as active nucleation platforms during the hydration process [40].

### 3.5. Defoaming Effect of GO on APC

**Figure 7:**
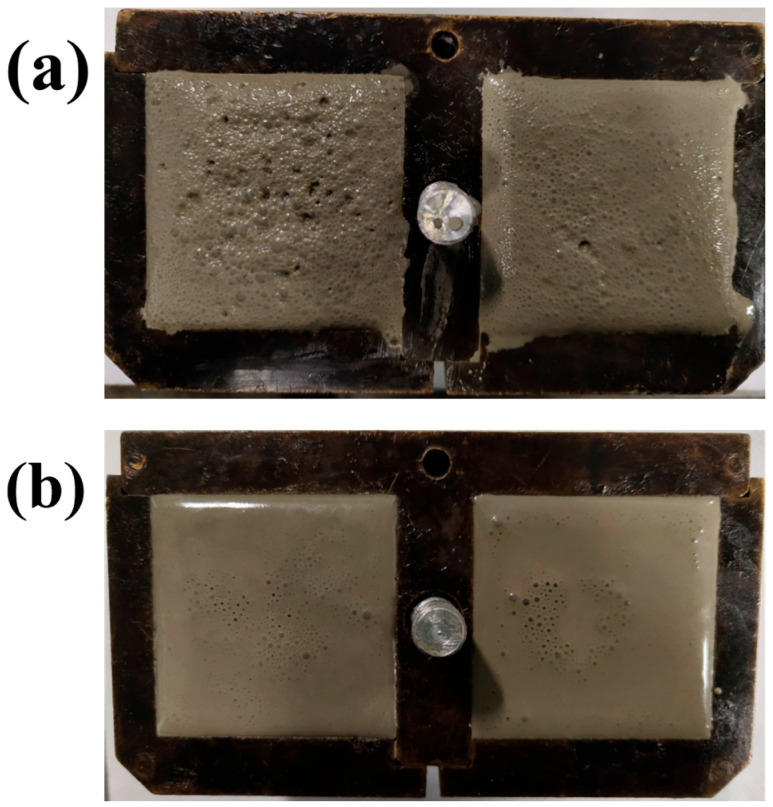
Images of fresh cement paste. (**a**) APC cement; (**b**) APC/GO cement.

During the cement mixing process, hydrophobic polymers with long branches have a tendency to trap air in the cement paste [41], forming harmful pores in the hardened cement and damaging its mechanical properties overall. In Figure 7a, many air bubbles were densely deposited on the fresh slurry surface because of the aggregation of APC polymers at the gas–liquid interface and its hydrophobic long chain, which trapped air. While the APC/GO composite eliminated the air bubbles efficiently (Figure 7b), well-dispersed GO can prevent the generation of bubbles. The defoaming ability of the APC/GO composite in the cement system was associated with the strong adsorption of the APC onto GO sheets, which restricted the polymer’s self-aggregation tendency and restrained its air-entrapping capacity. Meanwhile, surface tension of the dielectric composite changed and the stability of the bubbles was disrupted, leading to a reduction in the air content of the APC/GO-modified cement.

### 3.6. Pore Structure of Hardened Cement

**Figure 8:**
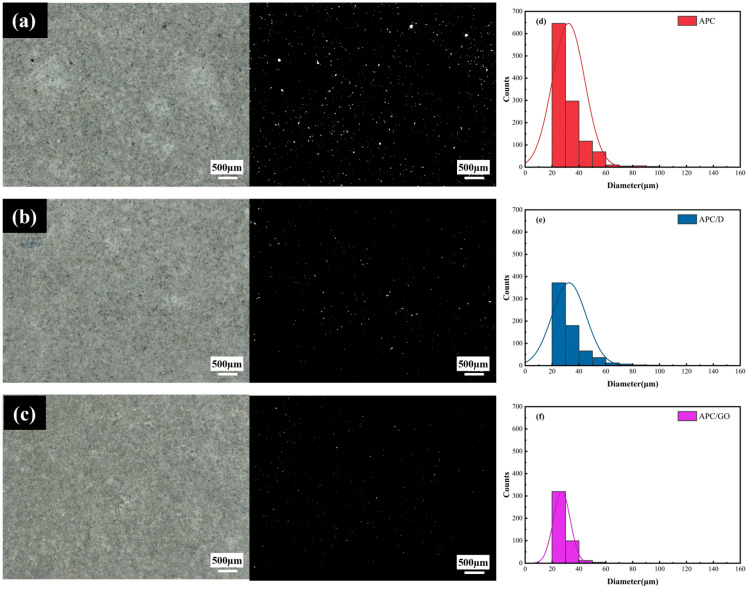
Cement composite microstructures. (**a**) APC, (**b**) APC/D, and (**c**) APC/GO, (**d**–**f**) Pore size distribution of APC-, APC/D-, and APC/GO-modified cement, respectively.

Microscopic images were obtained using a super depth of field (SDoF) system to verify the macropores in cement samples cured for 7 days. It was found that there were substantial macropores in the structure of the APC-modified cement cubes in Figure 8a. With the incorporation of defoamers, the number of air voids was reduced to some degree in the APC/D sample presented in Figure 8b due to the defoamer’s effects on the surface properties of the activated air bubbles [27]. As shown in Figure 8c, the addition of the APC/GO composite prevented and removed air voids more efficiently in the cement specimens.

To further validate the defoaming effect, a statistical histogram of pore size distribution was quantitatively generated using multiple captured microscopic images via an image analysis [42]. The frequencies of pore diameters in the range of 20 and 100 μm in the APC/GO sample were decreased significantly, as presented in Figure 8f, and were lower than in the APC- and APC/D-modified cement samples. The powerful attachment of the APC on GO sheets due to the electrostatic force would destroy the self-assembly of the APC at the gas–liquid interface to a large degree. Meanwhile, the well-dispersed GO displaced the polymer-foam-generating groups in a similar way to defoamers, thus diminishing the stability of the bubbles and removing them owing to its amphiphilic properties. Moreover, the nucleation effects of the well-dispersed APC/GO composite can improve the hydration degree of cement and reduce the pore diameter in cement composites.

**Figure 9:**
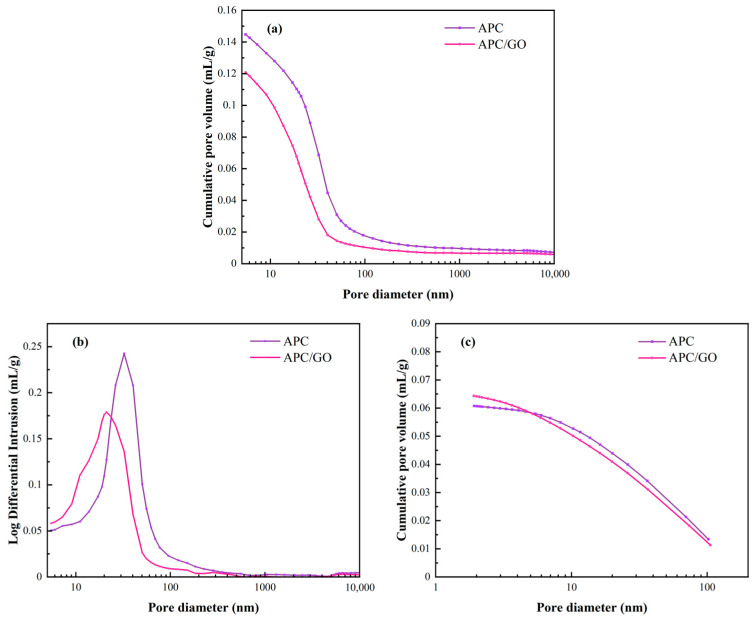
Pore structures of different cement samples. (**a**) Cumulative pore volume curves of cement specimens from MIP test; (**b**) log differential intrusion curves of cement specimens in MIP test; (**c**) cumulative pore volume curves of cement specimens from BET characterization.

To investigate the pore structure of cement at a microscale, MIP and BET assays were performed to measure micropores in the cement samples [43]. Figure 9a presents the cumulative pore volume curves of the specimens with APC and the APC/GO composite, respectively. A similar decrease in the macropores of the APC/GO sample was observed compared with the APC sample, which corresponds to the defoaming effect of GO on the APC discussed above. In Figure 9b, there was an apparent increase in the segmental porosity (<10 nm) of the APC/GO-modified cement composite, indicating that the incorporation of GO can facilitate the formation of gel pores in the cement matrix [44]. The BET characterization in Figure 9c exhibited similar densification effects of the APC/GO composite on cement. The well-dispersed GO can not only reduce macropores in cement but can also increase the proportion of gel pores originating from the formation of more hydrated products such as calcium silicate hydrate (C-S-H). The refining effect in the microstructure was ascribed to the defoaming effect of well-dispersed GO sheets decreasing the number of unfavorable large pores in the APC/GO cement sample. Another reason could be the abundant oxygen functional groups on the well-dispersed APC/GO sheets that could offer nucleation sites and promote cement hydration, thus contributing to a tighter pore structure [45]. To confirm the nucleation effects of the APC/GO composite, isothermal calorimetry characterization was conducted.

### 3.7. Development of Hydration Kinetics 

**Figure 10:**
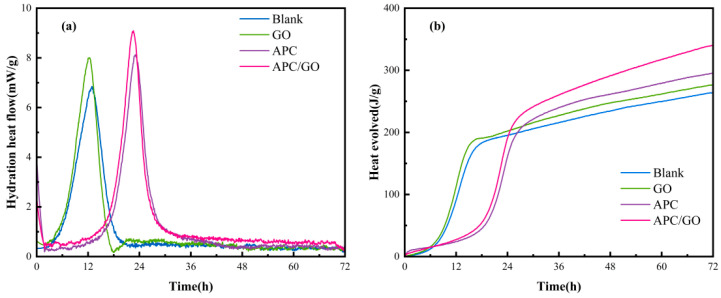
Hydration heat curves. (**a**) Heat evolution rate of cement pastes. (**b**) Cumulative heat of cement pastes.

Figure 10 presents the evolution of the heat flow and cumulative heat curves of cement specimens. The introduction of GO accelerated the hydration compared with pure cement, and its cumulative heat for 72 h was 277 J/g, which was only slightly higher than blank specimen (264 J/g). By introducing APC, the induction period of APC/cement specimen was significantly prolonged due to its retardation effect. The retardation effect was resulted from the chelation of Ca^2+^ with carboxylate groups in APC, which leads to a decrease in the Ca^2+^ concentration of pore solution and prevents the generation of hydrated products to some extent [46]. Besides, the adsorbed polymer acts as a coating, occupying the nucleation site and restraining ion and water diffusion at the cement and aqueous phase interface [47].

Compared with APC modified cement specimen, the incorporation of APC/GO caused critical hydration peak to be reached earlier and the peak intensity was 12.0% higher due to seed effect of GO sheets, that is, APC/GO promoted the alite (C_3_S) hydration and the formation of hydration products during the acceleration period. More importantly, the APC/GO enhanced the cumulative heat of cement paste by 36.90% than that of GO modified cement paste without APC (Figure 10b). This is because that the APC layer on GO plays the role of steric hindrance, inducing GO to be well dispersed in early hydration process, and the well-dispersed APC/GO could act as an active template to promote hydration more effectively. Apart from the effective dispersion of GO, the addition of APC would also destroy the cement flocculation structure, which released water molecules from the flocculates and further caused high cumulated heat in cement hydration.

**Figure 11:**
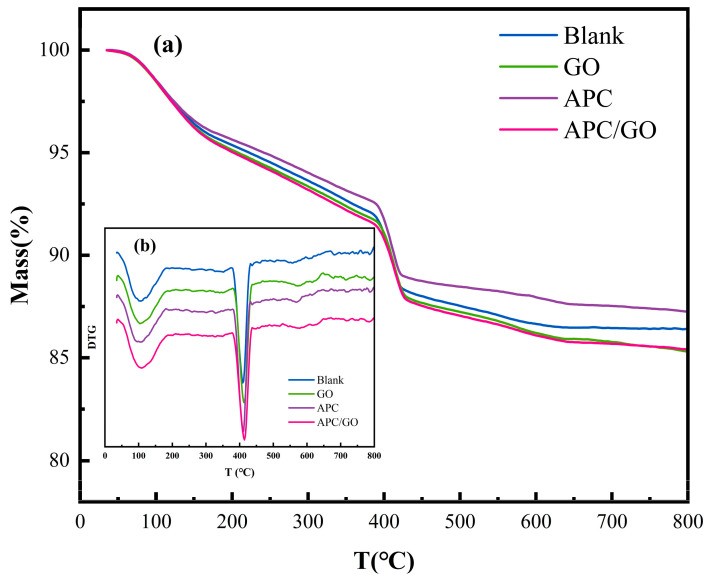
Thermal analysis curves of cement samples cured at 60 °C. (**a**) TG curves of cement pastes; (**b**) DTG curves of cement pastes.

To further analyze the composition of the hydration products, the hydrated product contents of different samples were evaluated using TGA measurements. Two significant endothermic peaks are visible on the TGA/DTG curves in Figure 11 which were caused by the dehydration of C-S-H/Aft and CH, respectively [48]. The quantity of CH was acquired from the TG curves using Equation (1) [37].

(1) $$CH\left(\%\right)={ML}_{CH}\left(\%\right)\frac{74}{18}$$

where *ML_CH_* (%) is the percent weight loss that happens when *CH* is dehydrated.

The calculated results are displayed in the Table 3. The proportions of C-S-H and CH in the GO-modified cement composites were higher than those in the blank sample. The improvements are viewed as proof of the GO speeding up the degree of hydration in the hydration process. As has been widely reported [22,49], the use of a superplasticizer in cement retards its early hydration process and postpones the growth of CH and C-S-H products, corresponding to the reduced CH and C-S-H contents in the APC samples. The addition of the APC/GO composite significantly increased the CH and C-S-H contents in the early hydration stage compared both with the APC sample and GO sample. The highest hydration product contents in the APC/GO cement sample were ascribed to the seed effects of the uniformly dispersed APC/GO composite in the cement. The well-dispersed APC/GO offered plentiful nucleation sites for hydrated products because it provided the largest surface area.

**Figure 12:**
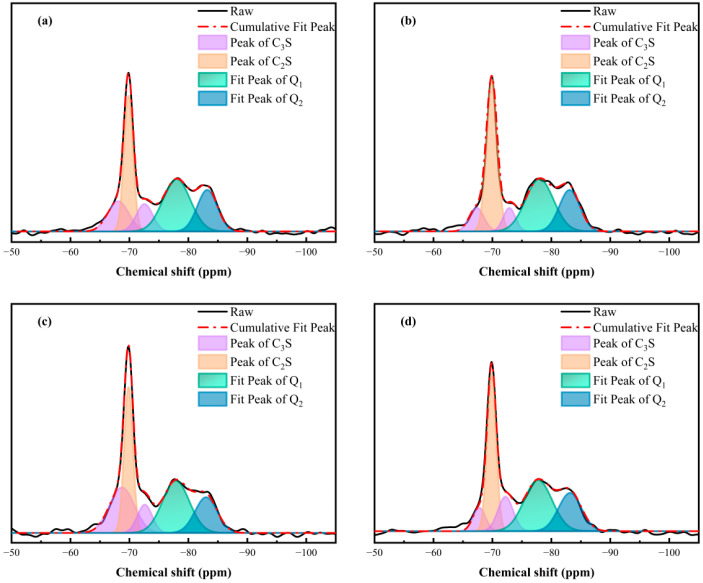
^29^Si MAS NMR spectra and deconvolution spectra of cement specimens curing at 60 °C. (**a**) blank sample, (**b**) GO, (**c**) APC, and (**d**) APC/GO.

Since C-S-H occupies the largest proportion of hydration products, its microstructure was further examined through ^29^Si NMR MAS [50]. As shown in Figure 12, Q^0^ phases concerning C_3_S and C_2_S and Q^1^ and Q^2^ phases for C–S–H gel were present in the spectra. The quantitative results calculated by deconvolution are shown in Table 4 [51]. The specimen containing GO showed a higher hydration degree compared with the blank specimen, revealing that the GO accelerated the hydration process of the silicate phases. After introducing the APC into the cement, the hydration degree (α) decreased from 49.71% (blank sample) to 43.65% (APC sample) because of its retardation effect. However, the α value in the APC/GO sample was raised up to 50.90%. The synergistic positive effects of the APC/GO composites on the hydration process lead to an increased hydration degree.

The hydration degree obtained via ^29^Si NMR MAS corresponds with the C-S-H content obtained via the TGA. The APC/GO sample also presented a slight increment in the MCL values, confirming a higher degree of C-S-H polymerization. The efficient nucleation effect of the well-dispersed APC/GO nanosheets was the primary factor in the high degree of C-S-H polymerization. Because of the hydrophilic functional groups and greater surface energy on the GO surface, it can be a template for the deposition and growth of C-S-H, facilitating the attachment of SiO_4_ tetrahedra to the dimers.

### 3.8. Mechanical Properties of Hardened Cement

**Figure 13:**
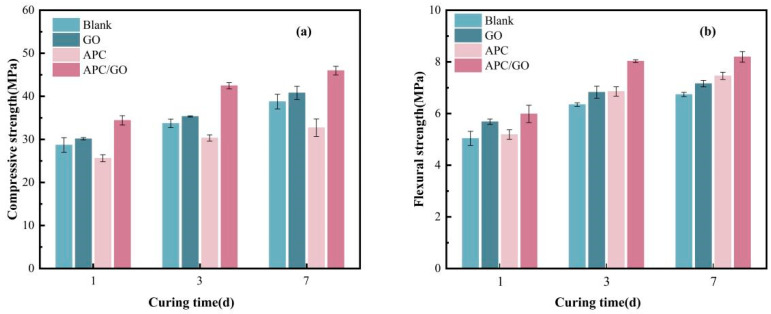
Compressive strength (**a**) and flexural strength (**b**) of cement samples.

The influence of the APC/GO composite on the compressive and flexural strength of the cement was analyzed. As shown in Figure 13a, the compressive strength of the APC specimen was extremely decreased in comparison with pure cement, which could be linked to the air- entraining ability of the APC dispersant in the process of mixing. The excess of air bubbles caused an unwanted porous structure inside the cement cube and thus impaired its compressive strength. Furthermore, the addition of the APC dispersant delays cement hydration, hinders the development of cement strength, and leads to a reduction in compressive strength.

The incorporation of the APC/GO composite significantly improved compressive strength, which had increased by approximately 40.53% and 18.58% at 7 days, respectively, compared with the APC-modified cement cube without GO and the blank samples. These results demonstrate the significant positive impact of the APC/GO composite on compressive strength, which could be contributed to the effective defoaming ability of the GO in the APC sample. The defoaming effect prevented the generation of air bubbles during mixing, which removed the negative impact of harmful macroscopic voids in the internal structure of the cement. Moreover, the increased hydration products on the well-dispersed GO refined the cement pore structure, which further served to enhanced its mechanical properties. The APC/GO specimen also exhibited enhanced strength compared to the GO sample without the APC, which proved that its good dispersion in the cement matrix was the primary factor contributing to the improved mechanical properties of the cement.

The flexural strength of cement samples modified by GO, an APC, and an APC/GO composite are exhibited in Figure 13b. The flexural strength of the APC/GO samples at 7d was enhanced dramatically by about 21.51% when compared to blank cement. This is because the well-dispersed GO nanosheets provide sufficient nucleation sites for hydrated products owing to their large specific surface area with abundant oxygenated groups, accelerating the hydration process and promoting the generation of a well-organized hydration crystal structure. The -COO- on the GO sheets can form ionic bonding with Ca^2+^ from the cement^10^, forming mechanical interlocking with hydration products and enhancing interfacial bonding between them [52]. Compared to the superior efficacy of the APC/GO composite in enhancing flexural strength, the enhancement exhibited by pure GO was found to be significantly limited. GO layers can easily be jointed or overlapped with each other as soon as cement is added to water, greatly decreasing its specific surface area and nucleation sites. The APC can prevent the self-agglomeration of GO and enable well-dispersed GO to explore more nucleation sites, thus promoting the growth of C-S-H and forming a densified microstructure in APC/GO samples. In addition, the APC polymer may form a polymer film [53] on hydrated crystals, and the continuous film in cement absorbs some external forces in the brittle fracture process.

## 4. Conclusions

A well-dispersed APC/GO toughening agent was successfully prepared by electrostatic self-assembly, and its defoaming effect and toughening mechanism were investigated. Several main conclusions were obtained as follows:

(1) The APC/GO composite has better dispersibility in a pore solution due to the steric hindrance effect of the APC’s side chains, which could protect the GO nanosheets from cross-linking with Ca^2+^.
(2) The powerful electrostatic attraction between GO and the APC restrained air entrainment due to the APC, allowing the well-dispersed GO to act as a defoamer. The microstructures of the cement composites verified that the APC/GO-modified cement composite had a lower macropore volume and higher segmental porosity compared to the APC cement, which are attributed to the defoaming effect and nucleation effect of the well-dispersed GO sheets.
(3) The well-dispersed APC/GO sample exhibited a 7-day compressive strength improved by 18.58% compared to a blank sample. The refined pore structure and higher hydration degree resulting from the nucleation effect are the main factors promoting the amelioration of the compressive performance of the cement.
(4) The flexural strength of the APC/GO sample increased by 21.5% at 7 days compared to a blank sample, mainly because a densified microstructure formed in the APC/GO samples and mechanical interlocking occurred between the GO and hydration products via crosslinking between -COO^−^ and Ca^2+^.

In summary, the modification of highly dispersed graphene oxide by an amphoteric polycarboxylate superplasticizer had a significant defoaming and toughening effect on oil well cement. GO can finely control the microstructure of cement blocks, increase gel pores, eliminate large pores, and surpass traditional defoamers in terms of its defoaming efficacy. Further detailed research will be conducted to unravel the mechanisms of nanoparticle defoaming. The addition of GO can comprehensively improve the mechanical properties of cement; thus, it is expected to be applied in construction cement and concrete.

## Figures and Tables

**Table 1:** The chemical composition of the cement.

Chemical Composition (wt.%)							
CaO	SiO_2_	Fe_2_O_3_	SO_3_	Al_2_O_3_	MgO	K_2_O	Others
70.08	14.71	6.235	2.88	2.547	1.05	0.835	1.663

**Table 2:** Mixing design for cement composites.

	w/c	APC (by Weight of Cement)	Defoamer	GO (by Weight of Cement)
Blank	0.44	-	-	-
GO	0.44	-	-	0.03%
APC	0.44	1%	-	-
APC/GO	0.44	1%	-	0.03%
APC/D	0.44	1%	(10% APC content)	-

**Table 3:** Calculation of C-S-H and CH content evolution in cement pastes through TGA.

Cement Sample	C-S-H (%)	CH (%)
Blank	2.39	16.25
GO	2.55	16.52
APC	2.20	15.63
APC/GO	2.66	16.56

**Table 4:** Mass percentages of silicon atoms in C–S–H.

Cement Sample	Relative Content (%)			α (%)	MCL
	Q^0^	Q^1^	Q^2^		
Blank	50.71	31.23	18.06	49.71	3.16
GO	49.77	31.72	18.51	50.23	3.17
APC	56.35	27.90	15.75	43.65	3.12
APC/GO	49.10	31.56	19.34	50.90	3.22

## Data Availability

Data are contained within the article.
